# 30-DAY READMISSION IN PATIENTS WITH CARDIOVASCULAR DISEASE: DO PATIENTS KNOW THEIR RISK BEFORE DISCHARGE?

**DOI:** 10.1093/geroni/igz038.3291

**Published:** 2019-11-08

**Authors:** Hanzhang Xu, Heather R Farmer, Bradi Granger, Matthew Dupre

**Affiliations:** 1 Duke University School of Medicine, Durham, North Carolina, United States; 2 Duke University, Durham, North Carolina, United States; 3 Duke University School of Nursing, Durham, North Carolina, United States

## Abstract

Cardiovascular disease (CVD) is the leading cause of disability and death in the United States, and older adults with CVD are at a high risk of readmission after discharge. This study examined whether patients’ perceived risk of readmission at discharge was associated with actual 30-day readmissions in patients with CVD. A standardized survey and electronic health records (EHR) were used to collect sociodemographic, psychosocial, behavioral, and clinical data on patients admitted to the Duke Heart Center (n=730). Prior to discharge, patients were asked their perceived likelihood of returning to the hospital for an unplanned/emergency visit within 30-days. Logistic regression models were used to examine all-cause 30-day readmission among patients who perceived low versus high readmission risk. Nearly 1-in-3 patients (31.4%) perceived high-risk of readmission at the time of discharge. Life stressors, poor self-rated health, and ADL limitations were associated with perceptions of high-risk. Patients who perceived high-risk had significantly higher subsequent readmissions compared with low-risk (23.3% vs. 15.6% p=0.016). Among patients who perceived low-risk of readmission, those who were widowed, had inadequate health literacy, and reported difficulty accessing care exhibited a higher likelihood of being readmitted. In those perceiving a high-risk, nonwhites and those with poor self-rated health, difficulty accessing care, and prior hospitalizations in the past year were significantly more likely to be readmitted. These findings have important implications for identifying CVD patients at high risk of readmission within 30 days after discharge, particularly older adults who may lack adequate resources (e.g., social support, literacy, access to care).

